# Pneumonitis resulting from radiation and immune checkpoint blockade illustrates characteristic clinical, radiologic and circulating biomarker features

**DOI:** 10.1186/s40425-019-0583-3

**Published:** 2019-04-24

**Authors:** Jonathan D. Schoenfeld, Mizuki Nishino, Mariano Severgnini, Michael Manos, Raymond H. Mak, F. Stephen Hodi

**Affiliations:** 1 Brigham and Women’s Hospital, Dana-Farber Cancer Institute, 450 Brookline Ave, Boston, MA 02215-5450 USA; 2 Dana-Farber Cancer Institute, Boston, MA USA

**Keywords:** Pneumonitis, Radiation., PD-1 inhibition., Biomarkers

## Abstract

**Background:**

Pneumonitis is a potential consequence of both lung-directed radiation and immune checkpoint blockade (ICB), particularly treatment with PD-1/PD-L1 inhibitors. Significant morbidity and mortality can result, and severe pneumonitis attributed to ICB precludes continued therapy. Thus, discriminating between radiation- and ICB- related pneumonitis is of importance for the increasing number of patients receiving both treatments. Furthermore, data are limited regarding the interplay between radiation- and ICB-induced lung injury, and which biomarkers might be associated with toxicity.

**Case presentation:**

We report longitudinal clinical and radiologic data, and circulating biomarkers in a melanoma patient treated with axillary radiation followed by ICB who developed consolidation and ground glass opacities (GGO) within the radiation field suggestive of radiation-pneumonitis followed by consolidation outside of the radiation field suggestive of ICB-related pneumonitis. Of note, symptomatic radiation-pneumonitis developed despite a low radiation dose to the lung (V20 < 8%), and ICB-related pneumonitis was limited to the ipsilateral lung, suggesting additive effect of radiation and ICB in the development of lung injury. Circulating biomarker analyses demonstrated increases in CXCR2, IL1ra and IL2ra that coincided with the development of symptomatic pneumonitis.

**Conclusions:**

These data highlight the imaging findings associated with radiation and ICB-related lung toxicity, and anecdotally describe a clinical course with circulating biomarker correlates. This information can help guide clinical evaluation and future research investigations into the toxicity of combined radiation immunotherapy approaches.

## Background

Pneumonitis develops in less than 5% of patients treated with PD-1/PD-L1 inhibitor ICB monotherapy. [1, 2] Many cases are relatively mild, and patients can resume ICB therapy following steroid treatment and resolution of symptoms. However, < 1% of cases are more severe [1], and patients can require prolonged treatment, require hospitalization, and be precluded from additional ICB treatment, even if this therapy is otherwise providing clinical benefit.

In addition to ICB, radiation therapy to the lung can also lead to an inflammatory pneumonitis generally treated with a lengthy course of corticosteroids in more severe cases. Rates of radiation pneumonitis vary significantly based on the amount of lung irradiated, as well as the dose of radiation that is delivered [3]. For example, in lung cancer patients, rates of grade 2 or higher pneumonitis were found to be 0% when the volume of the lung receiving 20 Gray (Gy) or higher was less than 22%, as compared to a 42% risk if the volume receiving 20 Gy or higher was greater than 40%. [4].

The rapid development of ICB across various indications including melanoma and non-small cell lung cancer (NSCLC) has resulted in an increasing number of patients treated with both ICB and lung-directed radiation, either concurrently or in close temporal proximity. Reassuringly, both retrospective and prospective data suggest that this combination is, in general, well tolerated [5–7]. More specifically, recent prospective studies do not suggest the combination of RT and ICB does not increase pneumonitis risk over each treatment individually [5, 7, 8]. However, these patients are at risk for both ICB- and radiation- mediated lung toxicity, and differentiating between the two can have important consequences relevant to clinical management such as impact on the decision to continue or restart ICB therapy. Attribution of toxicity also guides the evaluation of data in the clinical trial setting.

We report an instructive case of pneumonitis that developed in a patient with metastatic melanoma that developed following adjuvant axillary radiation that overlapped a portion of the right lung while the patient was treated with the PD-1 inhibitor nivolumab. Distinct radiologic features were initially consistent with radiation pneumonitis and subsequently evolved into findings outside of the radiation treatment field indicating ICB-related pneumonitis. Furthermore, manifestations of lung toxicity in this case were suggestive of an interaction between radiation and ICB-mediated toxicity, as the radiation induced pneumonitis developed at a relatively low radiation dose otherwise unlikely to result in symptomatic toxicity, and the ICB-related pneumonitis was limited to the ipsilateral right lung. Evaluation of circulating immune biomarkers revealed an increase in cytokine CXCL2, as well as IL1ra and IL2ra that tracked with the development of pneumonitis symptoms and then decreased with corticosteroid treatment.

## Case presentation

### Materials and methods

The study involved a melanoma patient treated with standard of care therapy who developed a spectrum of toxicity consistent with radiation and ICB-related pneumonitis. Blood was collected prospectively on an institutionally review board approved protocol. Clinical and radiologic data were subsequently collected retrospectively as allowed by the approved protocol. Clinical chest CT scans were obtained as standard of care and reviewed by a board-certified chest radiologist (M. Nishino).

Serum was isolated the day of blood collection (within 6 h) using centrifugation (3000 g, 10 min, 4 degrees Celsius) and these samples were stored at − 80 degrees Celsius and not thawed until the day of subsequent analysis. Cytokine levels were assessed with a custom Magnetic Luminex kit (Bio-Techne, Minneapolis, MN) including: TNFalpha, IL6, IL3, CCL7, MCP1, IL7, IL1ra, MIP1a, MIP1b, IL4, IL17A, IL2RA, IL5, IL8, GCP2, IL10, GROB, IL1B, IFNgamma, IL1a, GM-CSF, IL13, ENA78, C-CSF, IL12p70, IL15, and IP10. These concentrations were then analyzed using the FLEXMAP 3D Luminex System and quantified by Standard Curve extrapolation. All samples were tested in duplicate, according to manufacturer’s protocols.

### Clinical course

The patient was a 64-year-old fair-skinned man who was originally diagnosed with melanoma in 2013, at which time he had a lesion on his right lateral abdomen removed to reveal a BRAFv600 wildtype, Clark Level 3 melanoma that extended to 0.5 mm depth, with no ulceration and 1 mitosis / high powered field (HPF). The surgical margins were negative. He did not receive any additional treatment at this time.

In 2017, a cardiac MRI performed for viral myocarditis incidentally revealed enlarged right axillary lymph nodes. Ultrasound guided fine needle aspiration of these enlarged lymph nodes revealed melanoma, BRAFv600 wildtype. PET-CT demonstrated avidity within multiple axillary lymph nodes without clear evidence of other metastatic disease. MRI brain revealed changes thought to be more consistent with small past infarcts, also with no evidence of metastatic disease. Right axillary dissection performed in August 2017 revealed involvement of 13 of 31 axillary lymph nodes, the largest measuring 4.5 cm. There was significant extracapsular extension noted on pathology.

The patient was seen in multidisciplinary follow up, and recommendation was made for adjuvant radiation to the axillary bed given the risk factors for regional recurrence seen on pathology [9], followed by adjuvant PD-1 inhibition with nivolumab as supported by randomized clinical trial data [10].

**Fig. 1:**
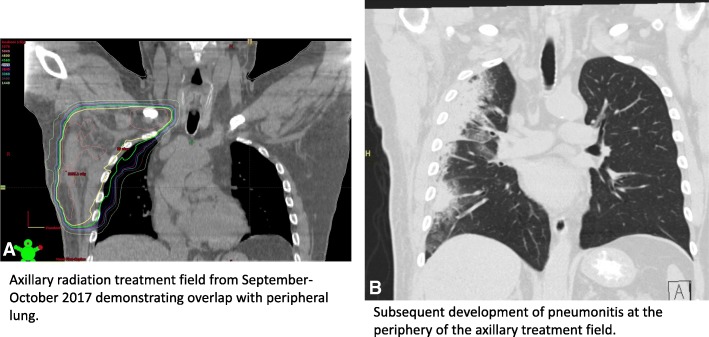
**a** (left) Axillary radiation treatment plan with radiation isodose curves overlaid demonstrates peripheral overlap with the lung **b** (right) Subsequent CT of the chest performed after starting ICB demonstrates peripheral consolidation and opacities at the periphery of the axillary treatment field

The patient received 5-field conformal radiation following computed tomography (CT)-simulation performed in the supine position with axillary fields defined using relevant preoperative imaging. Treatment was delivered using 6- and 10- MegaVolt (MV) photons using Novalis TX (Varian Medical Systems, Palo Alto, CA). Image guided radiation was delivered with daily kilovolt (kV) and weekly cone beam CT (CBCT) imaging. He received 48 Gy of right axillary radiation delivered over 20 fractions, completing in October 2017. Effort was made to minimize radiation dose to the lung to the extent possible (Fig. 1) with customized blocking and field design to accomplish this goal. The volume of the total right lung receiving 20 Gy was < 14% (V20 = 13.6%) and volume of the total lung receiving 20 Gy was < 8%. The volume of the right lung receiving 5 Gy was 77%. The patient tolerated treatment well, and PET-CT performed in January 2018 before starting ICB revealed no evidence of residual disease or lung injury (scan not shown).

**Fig. 2:**
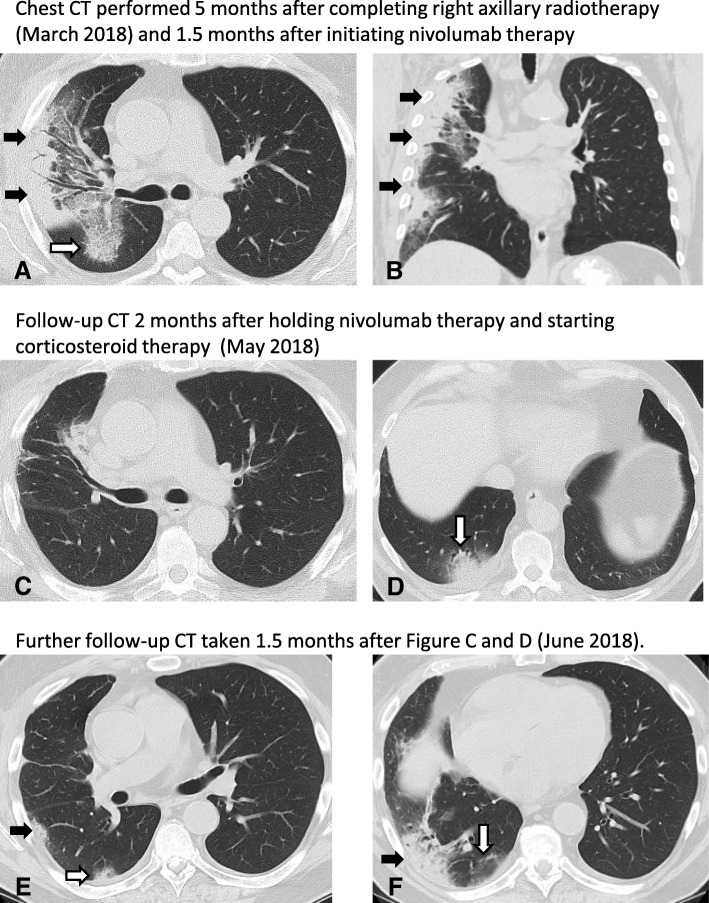
**a**, **b**. CT scan of the chest demonstrated peripheral curvilinear consolidative opacities predominantly in the right upper lobe (black arrows, **a**, **b**), accompanied by more central areas of ground glass opacities (GGO) and traction bronchiectasis. Most of the findings are within the radiation field, however, a focal area of GGO extended posteriorly into the superior segment of the right lower lobe (white arrow, **a**) which is outside of the radiation field. **c**, **d**. On a follow-up CT scan of the chest performed 2 months after A and B, previously noted peripheral consolidative opacities and GGO were mostly resolved, in response to corticosteroid therapy (**c**). However, a new focus of peripheral consolidation with surrounding GGO was noted in the right lower lobe (white arrow, **d**) outside of the irradiated lung field. **e**, **f**. Further follow-up CT taken 1.5 months after Fig. **c** and **d** demonstrated resolving peripheral consolidation that appeared on Fig. D noted as residual GGO (white arrow, **f**); however, additional new foci of peripheral consolidation with surrounding GGO are noted both outside of the radiation field (white arrows, **e**) and within the irradiated lung (black arrows, **e**, **f**)

In March of 2018, approximately 2 months after starting ICB, the patient presented with the gradual onset of progressive fatigue, shortness of breath, and a dry cough. He also developed diarrhea (2–3 times daily). Physical exam was notable for an oxygen saturation of 92–93% at rest and diminished breath sounds in the right lung. CT scan revealed peripheral curvilinear consolidative opacities in the right upper lobe accompanied by central areas of ground glass opacities (GGO) and traction bronchiectasis (Figure 1b and Fig. 2a and b). The majority of these changes were located within the edges of the prior radiation field that overlapped the lung as shown in Fig. 1. White blood cell count was within normal limits and there were no symptoms suggestive of infection.

The patient was briefly hospitalized, and he started treatment with corticosteroids at a dose of 1 mg/kg of IV solumedrol which was transitioned to oral prednisone. The corticosteroid treatment rapidly improved his symptoms and diarrhea. Nivolumab administration was discontinued and corticosteroids were slowly tapered. However, he developed increased symptoms of fatigue and shortness of breath in conjunction with the corticosteroid taper (at a dose of less than 0.2 mg/kg) approximately 2 months later in April 2018. His steroid dose was slightly increased and he was started on a course of levofloxacin following chest X-ray that raised concern for a right middle lobe infiltrate. Bronchoscopy with bronchioalveolar lavage was negative for an infectious source but did reveal mucopurulent fluid with 48% neutrophils and no atypical cells. CT scan performed at this time demonstrated marked resolution of the previously noted peripheral consolidative opacities and GGO (Fig. 2c), with a new focus of peripheral consolidation with surrounding GGO in the right lower lobe (Fig. 2d, arrow) which is outside of the radiation treatment field.

The patient’s symptoms briefly improved, but then worsened again in conjunction with another attempt at corticosteroid taper. Follow up CT scan demonstrated continued resolution of the peripheral opacities within the radiation treatment field as well as resolution of previously noted peripheral consolidation outside of the radiation treatment field (Fig. 2f, white arrow). However, there was a new focus of peripheral consolidation with surrounding GGO that was noted in the right lower lobe outside of the radiation treatment field in the right lung (Fig. 2e, white arrow). His corticosteroids were increased and his symptoms improved. Given ongoing difficulties tolerating corticosteroids (the patient was diabetic and was found to have significant episodes of hyperglycemia as well as oral candidiasis), the patient was treated with infliximab in late July 2018. His symptoms continued to improve and he was successfully tapered off corticosteroids without recurrence of respiratory symptoms. CT of the chest also normalized with resolution of GGO and nodular opacities that had been previously observed. He currently remains asymptomatic with no evidence of recurrent melanoma.

**Fig. 3:**
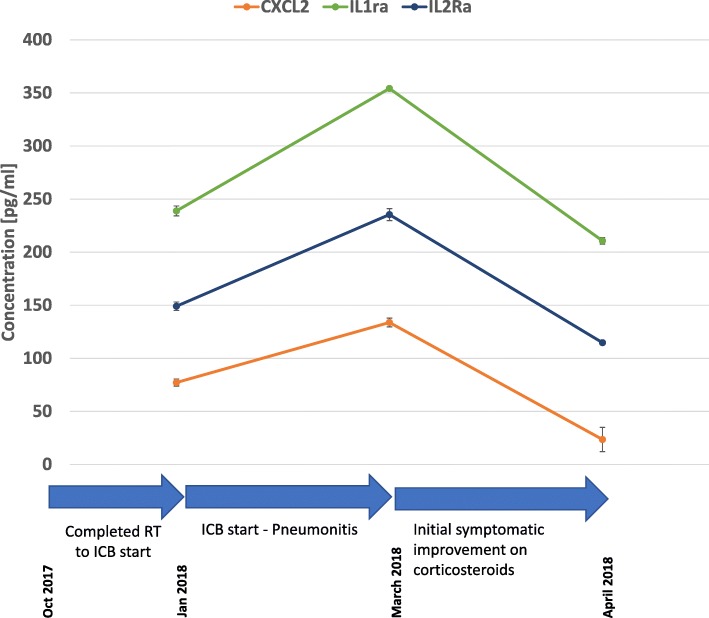
Timeline demonstrates change in circulating cytokines CXCL2, IL1ra, and IL2ra over the course of pneumonitis. Normal ranges obtained from testing pooled normal serum and the literature: CXCL2 47.8 pg/mL; IL1ra 1.3 pg/mL; IL2ra 1055 pg/mL^17^; Note: Second blood draw performed on 2/28/18 (at the time the patient developed symptoms consistent with pneumonitis. The CT scan of March 2018 consistent with pneumonitis was performed on 3/12/18

Research blood collection was analyzed from time-points: 1) prior to the initiation of ICB; 2) shortly prior to the initial development of symptoms of pneumonitis; and 3) during the initial steroid taper before symptoms recurred (Fig. 3). These analyses demonstrate a prominent increase in CXCL2, IL1ra, and IL2ra followed by a decrease in conjunction with corticosteroid treatment. Other cytokine levels remained relatively stable (data not shown).

## Discussion and conclusions

We describe the clinical course of a melanoma patient who had received axillary radiation followed by PD-1 inhibition with nivolumab for high-risk regionally metastatic disease. He developed symptomatic pneumonitis approximately 2 months after starting ICB, and 5 months after completing axillary radiotherapy. His symptoms improved with corticosteroid treatment, worsened when corticosteroids were tapered, and then finally resolved after treatment with the TNF-alpha-inhibitor infliximab.

The patient’s clinical course and radiologic findings are particularly notable because they illustrate the distinct manifestations of both radiation and ICB-related pneumonitis, as well as the potential interplay between the two processes. When the patient first developed symptoms, the CT findings of curvilinear consolidative opacities and GGO were predominantly localized to the radiation treatment field, suggestive of radiation pneumonitis. However, the degree and extent of lung involvement was somewhat more than what is usually expected from the dose of radiation exposure to the lung, considering the volume of the lung receiving greater than 20 Gy was relatively minimal, and the prescription dose was less than 50 Gy. Furthermore, attempt was made to limit the amount of lung receiving low dose radiation although 43% of the combined lungs and 77% of the ipsilateral lung received > 5 Gy. We do note that the risk of radiation-associated pneumonitis is likely dependent both on the maximum dose of radiation as well as the percentage of at risk tissue in the radiation field and low dose radiation exposure for which V5 is a surrogate. However, in this case, both the maximum dose to the lung and volume of lung irradiated were within parameters that are associated with a relatively low pneumonitis risk. It remains unclear whether higher and/or lower dose radiation parameters will be more or less predictive of pneumonitis in the setting of ICB, although the overall tolerability of combined radiation / ICB will likely make this a difficult question to address.

In contrast to the initial radiologic findings suggestive of radiation pneumonitis, the peripheral and lower lung consolidations eventually observed outside of the radiation field months later during the patient’s steroid taper is one of the most common radiographic patterns observed in the setting of PD-1 inhibitor-related pneumonitis [11–13]. However it is notable that this process was limited to the ipsilateral right lung that had received radiation while the left lung remained without evidence of pneumonitis. Finally, the subsequent pattern of lung injury that was observed consisting of waxing and waning consolidations in peripheral and lower lung distributions involving both irradiated and non-irradiated lung areas (distinct from the patterns observed initially) that occurred despite ongoing corticosteroid use indicate a more complex process as compared to pneumonitis related to either radiation or ICB alone, suggesting the possibility of effects from both immune-checkpoint blockade and radiation as an underlying mechanism.

In addition to the radiologic findings, we interrogated circulating cytokine levels over the course of treatment. We find that a few cytokines including CXCL2, IL1ra and IL2ra increase and then decrease in conjunction with the development of pneumonitis and subsequent treatment and before subsequent flares. Unfortunately, additional blood samples were not available at the time the patient developed additional symptoms in May/June 2018, nor after the patient’s treatment with infliximab, to investigate changes in more detail.

These results suggest the cytokines CXCL2, IL1ra and IL2ra should be evaluated in future clinical trial patients who develop radiation and/or ICB-related pneumonitis. CXCL2 is produced by monocytes and macrophages and signals as a chemoattractant for neutrophils and other immune cells that are active in inflammatory processes [14]. Both IL1ra and IL2ra were recently found to be part of gene signature that predict for toxicity in patients that were treated with ICB [15, 16]. It is also likely that additional cytokines and/or immune cell populations such as myeloid or other innate immune cells might play important roles in mediating effects of radiation / ICB.

Despite the frequency with which both radiation- and ICB- related pneumonitis occur, the pathophysiology remains unclear, and there is little data to suggest which clinical risk factors might be the most relevant. Preclinical evidence suggests that targeted radiation has immune stimulating effects, which could potentially increase the effectiveness of ICB, but may also add to toxicity and prompt immune-related adverse events. Clinical data has thus far been reassuring, with low rates of pneumonitis observed even in patients that receive the combination of ICB and lung directed radiotherapy [5–7]. However, it is challenging to identify rare idiosyncratic interactions as well as delayed effects. Toxicity localized to the radiation treatment field following subsequent ICB suggests a potential recall effect that has been described following higher dose lung-directed radiation for NSCLC followed by nivolumab therapy [17] and is a known effect of other antibiotic and chemotherapies. Finally, both radiation and ICB-related pneumonitis can have a protracted clinical course and relapse in conjunction with the tapering of corticosteroids [13], so it is unknown if the combination of radiation and ICB contributed to the severity of this case. It is reassuring that TNFalpha-inhibition, a more established treatment for ICB-induced toxicity, was clinically effective despite the potential contribution of radiation related lung injury in this case.

In summary, we have presented clinical and radiologic features of pneumonitis with biological correlates in a case of a melanoma patient treated with both radiation and ICB, who developed a particularly illustrative spectrum of lung toxicity. The radiologic findings demonstrated several different components that are individually more characteristic of either radiation or ICB-induced toxicity; however, when examined in combination and in clinical context, these findings raise the question whether initial lung injury from radiation can be exacerbated by ICB. Although it is important to reemphasize that prospective studies such as the PACIFIC trial [5] have demonstrated that the combination of lung directed radiation and ICB is not a high risk approach, further investigations are needed to elucidate the mechanisms underlying any potential interaction and identify potential clinical, radiologic and molecular predictors such as genetics or some underlying susceptibility, such as comorbidities or baseline inflammatory changes in the lung, that could lead to an increased risk of patients developing radiation induced pneumonitis exacerbated by immunotherapy.
